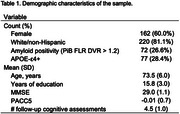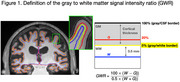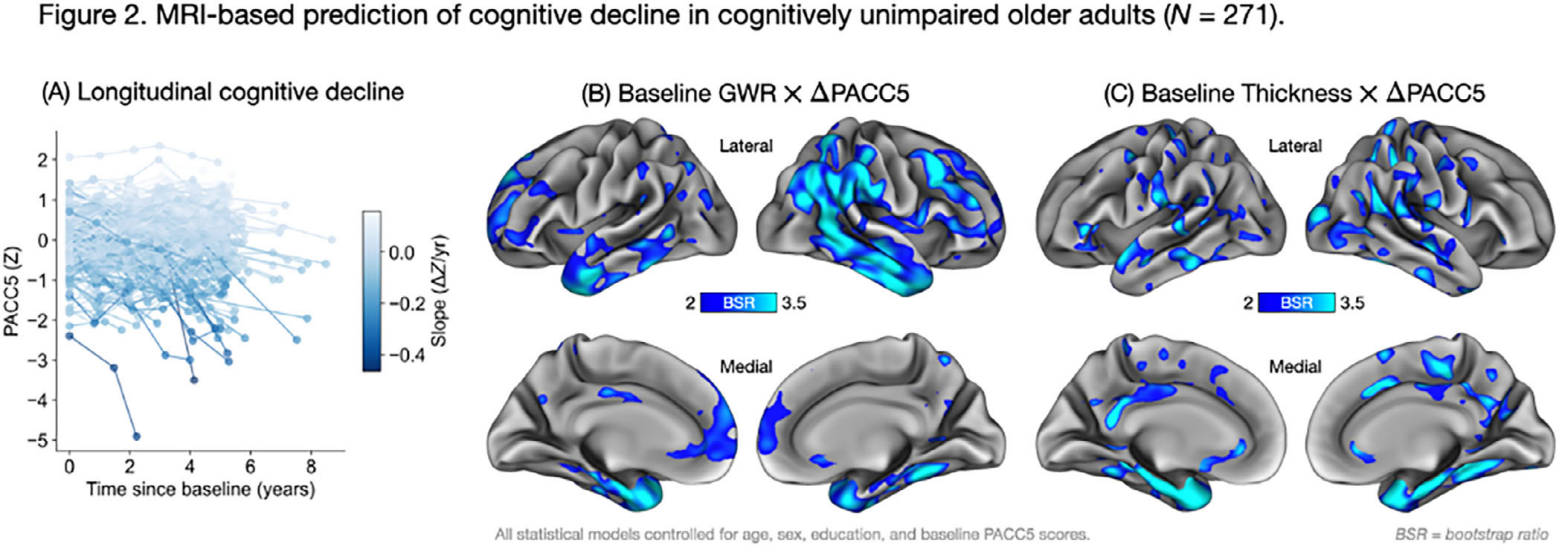# Gray to white matter signal ratio (GWR) as a novel MRI‐based biomarker of longitudinal cognitive decline in older adults with and without amyloid pathology

**DOI:** 10.1002/alz70861_108388

**Published:** 2025-12-23

**Authors:** Caitlin C Loxton, David H Salat, Brad C. Dickerson, Yuta Katsumi

**Affiliations:** ^1^ Massachusetts General Hospital, Boston, MA USA; ^2^ Frontotemporal Disorders Unit, Massachusetts General Hospital, Boston, MA USA; ^3^ Harvard Medical School, Boston, MA USA; ^4^ Athinoula A. Martinos Center for Biomedical Imaging, Department of Radiology, Massachusetts General Hospital, Harvard Medical School, Charlestown, MA USA

## Abstract

**Background:**

Microstructural properties of cortical gray matter are disrupted early in Alzheimer’s disease (AD), even before the onset of cognitive impairment. We and others have demonstrated across the symptomatic spectrum of AD that the gray to white matter signal intensity ratio (GWR), which quantifies the signal contrast between these tissue compartments in T1‐weighted MRI, may reflect pathological mechanisms that precede cortical atrophy. However, the utility of GWR for predicting longitudinal cognitive decline remains unclear. Here, we tested the hypothesis that decreased regional GWR at baseline—possibly indicative of early cortical microstructural changes—would predict a faster rate of cognitive decline among cognitively unimpaired older adults.

**Method:**

We analyzed baseline T1‐weighted structural MRI data collected using ADNI MPRAGE sequences from cognitively unimpaired individuals (CDR global scores = 0) part of the Harvard Aging Brain Study cohort (Table 1). MRI data were processed via FreeSurfer v7.1 to derive vertex‐wise estimates of GWR and cortical thickness (see Figure 1 for methods). Linear mixed‐effects models estimated the rate of change in Preclinical Alzheimer’s Cognitive Composite‐5 (PACC5) scores across participants. Multivariate partial least squares analysis was performed to identify the relationship between baseline GWR or thickness and PACC5 change.

**Result:**

PACC5 scores decreased on average by ‐0.034 standard deviation units annually over 4.5±1.0 years of follow‐up (Figure 2A). Reduced baseline GWR in widespread cortical areas (similar to the “AD signature” of atrophy defined in symptomatic patients) including the entorhinal cortex, ventral anterior temporal lobe, temporal pole, inferior parietal lobule, supramarginal gyrus, caudal and rostral lateral temporal cortex, and lateral and medial prefrontal cortex predicted subsequent decline in PACC5 scores (permutation testing *p* < .01; Figure 2B). Thinner cortex at baseline was also predictive of longitudinal PACC5 change (p < .01), although this effect was observed in fewer regions, most prominently in the medial temporal lobe (Figure 2C).

**Conclusion:**

In cognitively normal older adults, GWR at baseline is sensitive to early microstructural neurodegenerative change that predicts the rate of subsequent cognitive decline. Our findings support the utility of conventional T1‐weighted MRI for multi‐scale assessment of brain structural integrity, with potential applications in diagnosis, prognostication, and outcomes monitoring in neurodegenerative diseases.